# Role of Anticonvulsants in the Management of AIDS Related Seizures

**DOI:** 10.3389/fneur.2014.00010

**Published:** 2014-01-30

**Authors:** Batool F. Kirmani, Diana Mungall-Robinson

**Affiliations:** ^1^Department of Neurology, Epilepsy Center, Scott & White Neuroscience Institute, Texas A&M Health Science Center College of Medicine, Temple, TX, USA

**Keywords:** HIV, AIDS, antiepileptic drugs, antiretroviral agents

## Abstract

Seventy percent of the AIDS patients have neurological complications. Seizures are one of the complications and can occur at any stage. Seizures can be life-threatening and treatment with anticonvulsants is warranted. The therapeutic dilemma occurs in this case because of the interactions between the anticonvulsants, especially the first generation anticonvulsants, with antiretroviral agents resulting in significant side-effects including toxicity. The non-availability of second-generation anticonvulsants and cost constraints further limit the choices for the physicians. In this mini-review, we discuss the management of AIDS related seizures with emphasis on the drug–drug interactions between anticonvulsants and antiretroviral agents. We will also address the future directions and the need for prospective studies with second-generation anticonvulsants.

## Introduction

Neurological symptoms are commonly encountered in HIV patients, which range from meningitis, encephalopathy, and neoplasm to seizures (1, 2). New onset seizures were reported in AIDS patients in several studies (3–6). Holtzman and colleagues studied 100 cases of HIV patients and 18 out of 100 presented with seizures (3). Labar and Harden described generalized convulsions as most common seizures seen in 75% of the AIDS patients. Labar and Harden, in 13% of the patients, also reported generalized convulsive status epilepticus (7). Epilepsia partialis continua has also been reported in these HIV infected patients (8, 9). Bartolomei and colleagues described chronic focal myoclonus in AIDS patients (8). Ferrari et al. described HIV-associated progressive multifocal leukoencephalopathy (PML) presenting as epilepsia partialis continua (9). Pascual-Sedano and colleagues (10) reported partial seizures in 67% of the patients with an intracranial lesion.

The diagnosis of seizures in HIV patients includes neuroimaging, lumber puncture, and electroencephalogram (EEG). Harden et al. showed low amplitude EEG in AIDS dementia complex (11). Gabzuda et al. performed routine EEGs on a series of AIDS patients. The results showed generalized slowing in 38%, focal slowing in 19%, epileptiform activity in 6 and 37% had unremarkable EEGs (12).

In this mini-review article, we discuss the role of anticonvulsants in seizure management in AIDS patients.

### Treatment of AIDS related seizures with anticonvulsants in patients on antiretroviral therapy – benefits and pitfalls

Treatment with anticonvulsants in AIDS related seizures is necessary to prevent recurrent breakthrough seizures and to improve the quality of life of these patients.

There are several studies, which report the concomitant use of antiepileptic drugs (AEDs) in patients on antiretroviral therapy (ART). Birbeck et al. discussed the evidence-based guidelines from the International League against Epilepsy (ILAE) and the American Academy of Neurology (AAN) for antiepileptic drug selection for people with HIV/AIDS. The AAN and the ILAE formed a joint panel to evaluate the literature on concurrent usage of AEDs and antiretroviral drugs (ARVs). From 1950 to 2010, they queried six major databases and found 4,480 articles with potential data. These articles were reviewed first by their titles and abstract. The remaining 68 full articles were reviewed. Three Class III articles comprising 434,100,550 subjects identified a new onset seizure in 2.6–61% of HIV positive patients. In HIV patients who had not initiated ARV therapy, three class III studies of 255,101,272 patients indicated a 6.7–52.5% occurrence of peripheral neuropathy symptoms. This increased to 17–55% of HIV patients developing peripheral neuropathy symptoms after ARV initiation in two class III studies of 173, 2762 subjects. Patients receiving ritonavir/atazanavir may require an increase in lamotrigine to maintain levels, by as much as another 50% of the current lamotrigine dosage. Patients receiving valproic acid may require a reduction of zidovudine dose to maintain serum concentration of valproic acid. Patients may not require dosage adjustments if they are receiving valproic acid and efavirenz, raltegravir/atazanavir and lamotrigine, or raltegravir and midazolam.

The review of seven articles showing a correlation of enzyme-inducing antiepileptic drugs (EI-AEDs) and decreased viral suppression rates and subtherapeutic ARV levels led the panel to caution the use of EI-AEDs in patients with ARV therapy with protease inhibitors (PIs) or non-nucleoside reverse transcriptases (NNRTIs). When EI-AEDs need to be used, monitoring is recommended (13).

Siddiqi et al. conducted a literature review of the diagnosis and treatment of new onset seizures in patients with HIV. There are many potential causes of seizures in HIV positive patients, including infections, metabolic disturbances, medication side-effects, drug–drug interactions, and cerebrovascular disease. The etiology is unidentifiable in 28% of HIV positive patients. In the case of an acute symptomatic seizure due to an identifiable cause, AED treatment is recommended for 3–6 months with subsequent reassessment.

For chronic management of seizures, the choice of AED must take into consideration possible interactions with the patient’s ARV regimen. For patients treated with PIs and NNRTIs, studies recommend avoiding EI-AEDs, including primidone phenytoin, phenobarbital, and carbamazepine. However, in resource-limited settings, EI-AEDs are the most commonly used, and in some cases, are the only available options. Carbamazepine and phenytoin demonstrated a reduced half-life of the NNRTI nevirapine. While previous *in vitro* studies have shown valproic acid in HIV positive patients to stimulate HIV replication, *in vivo* studies have not shown this.

The authors state that when available, the recommended AEDs are levetiracetam followed by lacosamide, gabapentin, and pregabalin. All four are renally metabolized and do not interact with any AEDs or ARVs. Levetiracetam and lacosamide can be administered orally or intravenously, but levetiracetam has the additional benefit of being more moderately priced and does not need to be avoided in patients with second and third degree atrioventricular block, which is the case with lacosamide. Gabapentin and pregabalin can be administered only orally, and the costs range from moderate to expensive. In the event of medically refractory epilepsy, surgery may be an option for patients with focal brain lesions. Vagal nerve stimulators can be an option in poor surgical candidates but these require a higher level care center and greater costs (14).

Okulicz et al. conducted a case control study of HIV patients to determine the effect of EI-AEDs on serum antiretroviral levels. The EI-AEDs phenytoin, carbamazepine, or phenobarbital with concurrent use of a non-nucleoside reverse transcriptase (NNRTI; efavirenz or nevirapine) or a protease inhibitor (PI; lopinavir/ritonavir, atazanavir, or darunavir) were included. Ten study subjects were identified from the US Military HIV Natural History Study of 5,300 current and retired military members and beneficiaries with HIV aged at least 18 years. Patients were included if they had been on an ARV regimen for at least 6 months, on an EI-AED for at least 28 consecutive days during that period, and if serum EI-AED and ARV levels suggested medication compliance. Twenty-five control subjects with 30 overlap periods were identified as individuals that were not on an EI-AED (NEI-AED) and an ARV who met the inclusion criteria. In the study group, there were 16 periods of combined ARV/EI-AED use, most commonly for an unspecified seizure disorder (*N* = 5 of 10), focal epilepsy (*N* = 1), status epilepticus secondary to herpes simplex virus (HSV) encephalitis (*N* = 1), seizures secondary to PML (*N* = 1), trigeminal neuralgia (*N* = 1), or distal motor sensory neuropathy (*N* = 1). There were no statistically significant differences in demographics, with the age at diagnosis (32 years study group vs. 33 years control group), age at first AED/ARV overlap (42 vs. 47 years), female gender (10 vs. 12%), CD4 count at first AED/ARV overlap (377 vs. 397), and months of AED/ARV overlap (20 vs. 27). The EI-AED group had higher percentages than controls of overlap of intervals with one or more ARV intervals below *C*_min_ (37.5 vs. 23.3%; *p* = 0.124). This analysis excluded six below the reference range EI-AED levels in EI-AED/ARV overlap periods, there was a statistically significant difference when 60% of the EI-AED group AED/ARV overlaps with one or more ARV levels below *C*_min_ (vs. 23.3% *p* = 0.008). The authors concluded that there was a substantial impact of EI-AEDs on ARV drug levels (15).

In another study, Lee et al. conducted a retrospective chart review to investigate the impact of AED use in persons with HIV/AIDS and the *in vitro* effects of common AEDs on HIV replication and T cell proliferation. A search of the Southern Alberta Clinic database from January 2001 to May 2007 yielded 1,345 HIV positive patients in active treatment, of which 169 received AEDs. Sixty percent of these patients received AEDs for peripheral neuropathy, followed by 24% for seizure/epilepsy, 13% for mood disorders, and 2% for a movement disorder. The most commonly used AEDs were gabapentin, followed by valproate, carbamazepine, lamotrigine, phenytoin, and topiramate. AED-treated patients were divided into an “aviremic” group if they had received combined ART for at least 1 month with an undetectable plasma viral load before AED initiation, or “viremic” if they had a detectable viral load or had no previous ART prior to AED initiation. In a nested cohort of 55 aviremic patients receiving AEDs and ART, exposure to a sodium channel blocker (phenytoin, carbamazepine, lamotrigine) and calcium channel blockers (gabapentin/pregabalin) were associated after 12 months of therapy with increased CD4 T cell levels (*p* < 0.05), and had no change in CD8 T cell levels. The authors concluded that while patients had received high cumulative doses of AEDs with concurrent ART, there was a potential benefit to immune status and was comparatively safe (16).

Liedtke et al. conducted a literature review to investigate the interactions between AEDs and ARVs and their clinical significance. A PubMed search from 1966 to April 2003 using individual AED and ARV drug names with other key terms such as anticonvulsant, antiepileptic, and antiretroviral was conducted. The articles and scientific meeting abstracts were reviewed. Drug interactions between AEDs and PIs were described by six case reports. In patients receiving concurrent ritonavir and carbamazepine, there was a twofold to threefold increase in carbamazepine concentration resulting in toxicity. When carbamazepine and indinavir concurrent therapy was used, there was a loss of viral suppression. Decrease in phenytoin serum concentration with nelfinavir therapy was also reported in one patient. One patient with concurrent ritonavir and phenytoin had a 30% reduction in phenytoin serum concentration, while in another patient on the same combination there was no change. Two case reports of second generation AEDs and ARVs were included, which showed no CYP450 involvement. Lamotrigine was decreased in a patient receiving lamotrigine and ritonavir. A report of lamotrigine use with lopinavir/ritonavir, lamivudine, and stavudine resulted in a decline in viral load after 2 months of therapy and seizure freedom (17).

Romanelli et al. conducted a literature review of ARV and AED use in HIV positive patients. The authors did not include the methods of this search, key terms, number of articles found, or the review process of which articles to be included. Six epidemiological studies including 286 cases of new onset seizures reported that seizure etiologies were most commonly brain mass lesions (two of six studies), opportunistic infections (two of six studies), and idiopathic (two of six studies). In two of the studies that delineated between the different causes of opportunistic infections, cerebral toxoplasmosis was the most commonly reported (~30%). Six studies indicated the correlation of hypoalbuminemia increasing the free concentrations of highly protein bound drugs. AED examples were phenytoin, carbamazepine, clonazepam, and diazepam and ARV examples were delavirdine efavirenz, saquinavir ritonavir, nelfinavir, lopinavir, and amprenavir. Two cases of HIV-hypoalbuminemia reported increased free phenytoin concentrations in 21 patients and increased phenytoin and valproic acid levels, respectively. HIV hypergammaglobulinemia was noted in four cases to predispose patients to potentially life threatening hypersensitivity reactions. One of these reported that 14% of HIV positive patients receiving phenytoin had hypersensitivity reactions, including Stevens – Johnson syndrome. Finally, five studies included the possible correlation of valproic acid to stimulate *in vitro* HIV and cytomegalovirus (CMV) replication. However, in one included study, six of nine patients followed for 1–13 weeks did not show increase in viral loads. The authors advised avoidance or cautious use of phenytoin, if necessary (18).

Romanelli and colleagues also suggested in other literature that HIV positive individuals receiving AEDs such as VPA and phenytoin, commonly compete with other medications like trimethoprim/sulfamethoxazole (commonly used in HIV positive patients for prophylaxis against opportunistic infections). This may lead to increased free drug levels which in turn increases side-effects and toxicity. VPA has also been shown to stimulate the viral replication of HIV through the reduction of intracellular levels of glutathione and VPA has been reported to stimulate CMV replication. In patients receiving AEDs and ARVs, the authors suggest careful monitoring of viral load, disease progression, and AED serum levels. The authors conclude that additional controlled trials of pharmacokinetic and pharmacodynamics interactions are needed as well as *in vivo* studies of safety and efficacy of the use of AEDs in HIV positive individuals (19).

## Conclusion

The newer anticonvulsants, if available, should be used to minimize interactions with PIs and NNRTIs. The choices include levetiracetam, lacosamide, gabapentin, and pregabalin, which are renally excreted. Significant decrease in lamotrigine concentrations may be seen when used with ritonavir-based regimens. Viral load should be monitored closely if treated with valproic acid. Enzyme-inducing antiepileptic medications should be used with caution because of side-effects and drug–drug interactions with antiretroviral agents.

Limited data are available regarding the use of newer anticonvulsants because of a lack of availability in some countries and costs restraints in this patient subgroup. There is a need for larger prospective trials in order to provide more data about the role of newer anticonvulsants in HIV related seizures.

## Conflict of Interest Statement

The authors declare that the research was conducted in the absence of any commercial or financial relationships that could be construed as a potential conflict of interest.

## References

[1] Berger JR Simpson DM Neurologic complications of AIDS. In: Scheld WM Whitley RJ Durack DT, editors. Infections of the Central Nervous System. Philadelphia: Lippincott-Raven (1997). p. 255–71

[2] Britton CB Acquired immunodeficiency syndrome. In: Rowland LP, editor. Merritt’s Textbook of Neurology. Baltimore: Williams & Wilkins (1995). p. 179–93

[3] Holtzman D Kaku DA So YT New-onset seizures associated with human immunodeficiency virus infection: causation and clinical features in 100 cases. Am J Med (1989) 87:173–7 10.1016/S0002-9343(89)80693-X 2757058

[4] Aronow H Feraru E Lipton R New-onset seizures in AIDS patients: etiology, prognosis, and treatment. Neurology (1989) 39(Suppl 1):4282927656

[5] van Paesschen W Bodian C Maker H Metabolic abnormalities and new-onset seizures in human immunodeficiency virus-seropositive patients. Epilepsia (1995) 36:146–50 10.1111/j.1528-1157.1995.tb00973.x 7821271

[6] Pesola GR Westfal RE New-onset generalized seizures in patients with AIDS presenting to an emergency department. Acad Emerg Med (1998) 5:905–11 10.1111/j.1553-2712.1998.tb02820.x 9754504

[7] Labar DR Harden C Infection and Inflammatory Diseases. In: Engel J Jr Pedley TA, editors. Epilepsy: A Comprehensive Textbook. Philadelphia, PA: Lippincott-Raven (1997). p. 2587–96

[8] Bartolomei F Gavaret M Dhiver C Gastaut JA Gambarelli D Figarell-Branger D Isolated, chronic, epilepsia partialis continua in an HIV-infected patient. Arch Neurol (1999) 56:111–4 10.1001/archneur.56.1.111 9923770

[9] Ferrari S Monaco S Morbin M Zanusso G Bertolasi L Cerini R HIV-associated PML presenting as epilepsia partialis continua. J Neurol Sci (1998) 161:180–4 10.1016/S0022-510X(98)00281-0 9879702

[10] Pascual-Sedano B Iranzo A Marti-Fabregas J Domingo P Escartin A Fuster M Prospective study of new-onset seizures in patients with human immunodeficiency virus infection: etiologic and clinical aspects. Arch Neurol (1999) 56:609–12 10.1001/archneur.56.5.609 10328257

[11] Harden C Daras M Tuchman A Koppel BS Low amplitude EEGs in demented AIDS patients. Electroencephalogr Clin Neurophysiol (1993) 87:544–6 10.1016/0013-4694(93)90174-T 7687954

[12] Gabuzda DH Levy SR Chiappa KH Electroencephalography in AIDS and AIDS-related complex. Clin Electroencephalogr (1988) 19:1–6 3396199 10.1177/155005948801900103

[13] Birbeck GL French JA Perucca E Simpson DM Fraimow H George JM Evidence-based guideline: antiepileptic drug selection for people with HIV/AIDS: report of the Quality Standards Subcommittee of the American Academy of Neurology and the Ad hoc Task Force of the Commission on Therapeutic Strategies of the International League Against Epilepsy. Neurology (2012) 78:139–45 10.1212/WNL.0b013e31823efcf8 22218281 PMC3466673

[14] Siddiqi O Birbeck GL Safe treatment of seizures in the setting of HIV/AIDS. Curr Treat Options Neurol (2013) 15:529–43 10.1007/s11940-013-0237-6 23657845 PMC3744057

[15] Okulicz JF Grandits GA French JA Perucca E George JM Landrum ML The impact of enzyme-inducing antiepileptic drugs on antiretroviral drug levels: a case-control study. Epilepsy Res (2013) 103:245–53 10.1016/j.eplepsyres.2012.07.009 22835761 PMC3508295

[16] Lee K Vivithanaporn P Siemieniuk RA Krentz HB Maingat F Gill MJ Clinical outcomes and immune benefits of anti-epileptic drug therapy in HIV/AIDS. BMC Neurol (2010) 10:44 10.1186/1471-2377-10-44 20565780 PMC2902446

[17] Liedtke MD Lockhart SM Rathbun RC Anticonvulsant and antiretroviral interactions. Ann Pharmacother (2004) 38:482–9 10.1345/aph.1D309 14970370

[18] Romanelli F Pomeroy C Concurrent use of antiretrovirals and anticonvulsants in human immunodeficiency virus (HIV) seropositive patients. Curr Pharm Des (2003) 9:1433–9 10.2174/1381612033454676 12769723

[19] Romanelli F Jennings HR Nath A Ryan M Berger J Therapeutic dilemma: the use of anticonvulsants in HIV-positive individuals. Neurology (2000) 54:1404–7 10.1212/WNL.54.7.1404 10751246

